# Comparison of the accumulation of macro- and microelements in the bone marrow and bone of wild and farmed red deer (*Cervus elaphus*)

**DOI:** 10.1186/s12917-021-03041-2

**Published:** 2021-10-10

**Authors:** Katarzyna Tajchman, Aleksandra Ukalska-Jaruga, Marek Bogdaszewski, Monika Pecio, Pawel Janiszewski

**Affiliations:** 1 Department of Animal Ethology and Wildlife Management, Faculty of Animal Sciences and Bioeconomy, University of Life Sciences in Lublin, Akademicka 13, 20-950 Lublin, Poland; 2 Department of Soil Science Erosion and Land Protection, Institute of Soil Science and Plant Cultivation, State Research Institute, Czartoryskich 8, 24-100 Puławy, Poland; 3 Institute of Parasitology of the Polish Academy of Sciences, Research Station in Kosewo Górne, 11-700 Mrągowo, Poland; 4 Department of Fur-bearing Animal Breeding and Game Management, University of Warmia and Mazury in Olsztyn, Oczapowskiego 2, 10-719 Olsztyn, Poland

**Keywords:** Availability of elements in bone marrow, Diverse living conditions, ICP-MS, Mineral composition of bone and marrow

## Abstract

**Background:**

The cells of the entire body, including the skeletal system, especially of young animals, may derive from the bone marrow in which they multiply. Therefore, it is important to assess whether the diet and quality of life of deer have a significant impact on the elemental composition of bone and bone marrow, which can directly affect their health and growth. The aim of this study was to determine the concentrations of macro- (Ca, calcium, P, phosphorus, Mg, magnesium, K, potassium, Na, sodium) and microelements (Li, lithium, Cr, chromium, Mn, manganese, Co, cobalt, Cu, copper, Zn, zinc, Se, selenium, Mo, molybdenum, and Sn, tin) accumulated in the bone marrow and bones of deer (*Cervus elaphus*). The study was carried out on 15 young stags divided into two groups: farmed and wild animals. The concentrations of macro- and microelements were analysed using the inductively coupled plasma mass spectrometry technique. This research expands our knowledge on this topic, which so far has not been extensively studied.

**Results:**

The mean content of K, Na, Zn and Se in the bone marrow of farmed animals was significantly higher than in wild deer, whereas the mean content of Ca, P, Mg, K, Na and Li in the bones was higher in wild animals than in farmed individuals (*p* < 0.05). In addition, the mean concentration of Cr, Mn, Cu, Se and Mo in the bones of the analysed animals differed significantly (*p* < 0.05) and was higher in the farmed deer. The mean concentration of Se in the bone marrow of wild deer decreased with the increase of the body weight (*p* < 0.05). In turn, the mean content of Mn in the bone marrow and of Mo in the bones of the animals was significantly positively correlated with the animals’ body weight (*p* < 0.05).

**Conclusions:**

The obtained results indicated different levels of micro- and macro-components in the body of farmed and wild deer, though without clear and strong variations. Generally, the higher level of macronutrients in the bones of wild deer may be related to the higher physiological importance of these minerals for life activities in the natural environment and to the limited supply of balanced food. On the other hand, the higher levels of microelements in the tissues of farmed animals may result from their significantly better nutritional status in the first year of life, achieved through appropriate nutrition as well as diet supplementation of adult females.

**Supplementary Information:**

The online version contains supplementary material available at 10.1186/s12917-021-03041-2.

## Background

Macroelements (Ca, calcium; P, phosphor; Mg, magnesium; K, potassium; and Na, sodium) as well as microelements (Li, lithium; Cr, chromium; Mn, manganese; Co, cobalt; Cu, copper; Zn, zinc; Se, selenium; Mo, molybdenum; and Sn, tin) exert a huge impact on the proper development of the animal skeletal system. Their deficiency may weaken or even inhibit animal growth and development, deteriorate the immune system, delay sexual maturity, and lead to bone fragility, e.g., osteochondrosis and osteoporosis [1–3]. Therefore, their appropriate concentration is extremely important, especially in young animals. This is true also for deer, which have specific nutrient requirements during antler development. Previous studies have demonstrated that the early growth of fawns determines their future reproductive success and development [4–14]. Favourable environmental conditions ensure the good health of young deer and accelerate maturation up to 2 months earlier than the standard age (at 14–16 months) [15]. In contrast, adverse conditions can significantly delay maturation even to the third or fourth year of life. Cervids are deemed to have attained maturity when they reach 65–70% of the average adult weight, which is determined by the availability and quality of food [16–20]. Moreover, the availability of macro- and microelements is particularly important, as these animals suffer from cyclic physiological osteoporosis during antler growth [10–14]. Some micronutrients, such as Zn or Se, may interfere with the absorption of harmful compounds, which seems to be particularly beneficial for deer [21]. It has been shown that animals may accumulate quite high concentrations of some toxic elements [22]; therefore, it is important to determine the accumulation of macro- and microelements. However, deer are able to modulate mineral absorption according to their needs and reduce this process when their needs have been satisfied [23–25]. Wild-living deer, which, unlike farmed cervids, have the ability to move and feed on various species of forest and crop plants, can compensate for the lack of nutrients necessary for normal growth and metabolic processes [26–32]. Farmed deer are grazed on pastures that have a limited variety of grasses and herbaceous plants, which may prevent a balanced diet [33]. In particular, in winter, some roughage is commonly provided to preserve the rumen function and slightly increase the protein supply, but minerals are rarely added. Thus, diet supplementation may help to maintain the animals in good health, but does not necessarily ensure a proper mineral supply.

The major minerals found in bone are calcium and phosphorus in the form of an insoluble salt called hydroxyapatite. Hydroxyapatite crystals lie adjacent and bound to the organic protein matrix. Magnesium, sodium, potassium and citrate ions are also present, conjugated to hydroxyapatite crystals rather than in distinct crystals of their own [34]. In deer’s body, about 90% of phosphorus occurs in the form of calcium phosphate; thus, it is extremely important to maintain a phosphate–calcium balance in the body, which depends to a large extent on the appropriate ratio of calcium to phosphorus in the food. Zinc, copper and manganese are components of the enzymes that synthesize the bone matrix. Among these elements, Zinc has a great impact on the growth of the skeletal system, as it determines the proper mineralization and functioning of osteoblasts. Therefore, its deficiency can lead to a dysfunction in the maturation of the skeletal system. Copper and manganese act synergistically with calcium, while potassium improves the calcium balance by reducing the excretion of calcium in the urine, which prevents its resorption (release) from the bones [35].

Bones constitute the main part of the skeleton, have a structural function, and are also involved in organ protection, locomotion, muscle activity, and load bearing. Moreover, bones are formed in living organisms in their first stage of growth and development, and macro- and micronutrients are important building components of bones [36]. Antlers are a specific type of bone growing from the frontal bone on the head of male deer, devoid of horny substance. They grow fast and are shed every year; therefore, they must gain weight in a very short time [37, 38]. Thus, while the chemical composition of antlers may reflect an animal’s diet in the recent past, internal bones contain information on its entire feeding cycle. Moreover, the effect of macro- and microelements on bone properties is considerably more important than the effects on antlers. Dietary macro- and micronutrient deficiencies in deer can have serious consequences, e.g., bone fracture, which can be life threatening, in contrast to an antler fracture [39]. Therefore, a long-term availability of minerals in the diet plays a very important role and can affect the survival and development of deer. This also emphasises the significant role of bone as a store of minerals [40].

Most macro- and microelements are highly important for the functioning of the circulatory system in animals. They ensure the normal division of red blood cells and are involved in the formation of bone marrow, which can be a mineral reservoir for other tissues, thus preventing osteoporosis. Bone tissue as well as many other tissues in the body (especially in newborns and young animals) are built of cells originated in the bone marrow, consisting largely of haematopoietically active tissue composed of relatively small amounts of adipose tissue. Billions of cells per kilogram of body weight are produced each day in the bone marrow. Hence, the concentration of macro- and microelements in the bone marrow may have an impact on the entire organism and on the accumulation of the mentioned elements. Therefore, the aim of our study was to determine the concentration of macro- (Ca, P, Mg, K and Na) and microelements (Li, Cr, Mn, Co, Cu, Zn, Se, Mo and Sn) accumulated in the bone marrow and bones of deer (*Cervus elaphus*) and compare their concentration in these tissues between wild and farmed animals.

## Methods

### Experimental design

The study was conducted on the same animal population as that described in Tajchman et al. [22]. Briefly, two groups of red deer living in different habitats were analysed: the first group of animals comprised farmed deer (*n* = 6), while the second group consisted of wild (*n* = 9) deer. The analysis included 15 male deer in their first year of life, i.e., 6–7-month-old, since young stags are usually culled in the breeding process, while hinds are intended for reconstruction of the herd. The farmed deer were bred at the Research Station of the Institute of Parasitology, Polish Academy of Sciences, Kosewo Górne in Poland. The breeding system was based on rotational pasture within plots with an area and density recommended by DEFRA [41], FEDFA [42], and Mattiello [33]. The study involved fawns born in a natural way, during the grazing period, which lasts from April to November in Poland. At the beginning of their lives, they were fed by hinds with milk, and later they ate the vegetation available on the pasture. Older animals in the winter period (from December to March) were fed ad libitum with grass haylage or hay with average nutritional value, concentrated feed based on compressed oats and protein supplements in the form of rapeseed, soybean concentrates (Eco-pasz, Poland) and Josera Phosphoreimer multi-ingredient licks (Josera, Poland) (Table 1).

**Table 1:** Composition of Josera Phosphoreimer multi-ingredient licks (Josera, Poland)

Components		Content (in 1 kg)
Ca	%	5.00
P	%	10.00
Na	%	7.00
Mg	%	7.50
Ca / P		0.5:1
Vitamin A	j.m.	650,000.00
Vitamin D3	j.m.	120,000.00
Vitamin E	mg	1500.00
Zn (as zinc oxide)	mg	8000.00
Mn (as manganese chelate of glycine hydrate)	mg	4000.00
Mn (as manganese (II) oxide)	mg	4000.00
Cu (as copper sulphate pentahydrate)	mg	1200.00
I	mg	100.00
Co	mg	22.00
Se (as sodium selenite)	mg	40.00

The second group of animals comprised wild individuals living in the area of Strzałowo Forest District in the immediate vicinity of the deer farm.

### Sampling

The body weight of the farmed and wild animals was measured as described in Tajchman et al. [22]. Bones and bone marrow from all animals were collected in the fall, in November 2019. Fresh bones were carefully opened with a dental titanium drill to prevent contamination of the bone marrow, then collected and deep-frozen (at 30 °C). After removal of the yellow marrow, the middle part of the bones (diaphysis, compact bone) were dried and fragmented by a dental drill.

### Analysis of microelement concentrations in bone and bone marrow of wild and farmed red deer

The analysis of macro- and microelement concentrations was conducted using inductively coupled plasma mass spectrometry (Agilent quadrupole 7500 CE ICP-MS). The extracts were prepared in concentrated nitric acid by microwave digestion. A blank sample and certified reference material (NIST1400 and CRM028-050) were included in the analyses for quality control of the entire analytical process. The basic validation of the parameters included the recognition of recovery, ranging from 90 to 97%, and precision, defined as a relative standard deviation < 3%. The limit of detection (LOD) was from 0.007 mg kg^− 1^ to 0.099 mg kg^− 1^.

### Statistical analysis

All results were expressed as the mean, with standard deviation calculated for at least three repetitions (*n* = 3). The distribution of the studied variables was measured by the Shapiro–Wilk test and, on this basis, further statistical analyses were carried out. Thus, statistical differences between the concentrations of individual macro- and microelements in deer bones and bone marrow were analysed using the Student’s t-test and the Mann–Whitney U test. The dependencies between variables were expressed by the r-Pearson correlation coefficient and the Spearman rank correlation coefficient. Moreover, the Student’s t-test and the Wilcoxon test were used for statistical evaluation of normally and non-normally distributed variables, respectively. All relationships were assessed according to the significance level at *p* < 0.05 within the confidence interval of 95%. The Statistica 9.1 software was used to evaluate the obtained results.

## Results

### The concentration of macro- and microelements accumulated in the bone marrow and bones of red deer

The accumulation of most macro- and microelements in the bone marrow and bones of both studied groups of animals was demonstrated. The mean concentration of Ca in the bone marrow was at the level 1192.725 mg kg^− 1^ and 2462.461 mg kg^− 1^, P 770.061 mg kg^− 1^ and 1520.283 mg kg^− 1^, Mg 34.972 mg kg^− 1^ and 72.681 mg kg^− 1^, K 91.221 mg kg^− 1^ and 294.103 mg kg^− 1^, Na 369.312 mg kg^− 1^ and 2078.073 mg kg^− 1^, Cr 0.038 mg kg^− 1^ and 0.079 mg kg^− 1^, Mn 0.008 mg kg^− 1^ and 0.027 mg kg^− 1^, Cu 0.216 mg kg^− 1^ and 0.487 mg kg^− 1^, Zn 1.309 mg kg^− 1^ and 2.296 mg kg^− 1^, Se 0.007 mg kg^− 1^ and 0.036 mg kg^− 1^, Mo 0.001 mg kg^− 1^ and 0.018 mg kg^− 1^, Sn 0.102 mg kg^− 1^ and 0.166 mg kg^− 1^ in wild and farm animals, respectively. The average Ca content in the bones was at the level 320,545.995 mg kg^− 1^ and 273,469.050 mg kg^− 1^, P 110062.456 mg kg^− 1^ and 92,739.045 mg kg^− 1^, Mg 6103.294 mg kg^− 1^ and 4537.109 mg kg^− 1^, K 551.586 mg kg^− 1^ and 434.745 mg kg^− 1^, Na 8606.449 mg kg^− 1^ and 7082.525 mg kg^− 1^, Li 1.421 mg kg^− 1^ and 0.232 mg kg^− 1^, Cr 0.131 mg kg^− 1^ and 4.148 mg kg^− 1^, Mn 0.788 mg kg^− 1^ and 1.388 mg kg^− 1^, Co 0.065 mg kg^− 1^ and 0.091 mg kg^− 1^, Cu 0.054 mg kg^− 1^ and 0.115 mg kg^− 1^, Zn 63.343 mg kg^− 1^ and 59.249 mg kg^− 1^, Se 0.003 mg kg^− 1^ and 0.006 mg kg^− 1^, Mo 0.027 mg kg^− 1^ and 0.398 mg kg^− 1^ in wild and farm deer, respectively. The content of Li in the bone marrow of all animals was below the limit of detection likewise the level of Co in the bone marrow in the wild animals and Sn in the bones of the farmed deer (Table 2).

**Table 2:** Comparison of the microelements concentrations in the bones and bone marrow between the wild and farmed red deer

Analyzed parameters			Wild red deer		Farm red deer		t^**a**^/Z^**b**^	p
			M	SD	M	SD		
Bone marrow	Ca	mg/kg	1192.725	919.792	2462.461	2277.757	-1.519^a^	0.152
	P		770.061	516.075	1520.283	1111.409	-1.527^a^	0.174
	Mg		34.972	15.076	72.681	42.140	15.000^b^	0.181
	K		91.221	23.598	294.103	282.351	-2.186^a^	0.047*
	Na		369.312	73.577	2078.073	2196.267	2.000^b^	0.001*
	Li		<LOD	<LOD	<LOD	<LOD	–	–
	Cr		0.038	0.052	0.079	0.079	16.000^b^	0.223
	Mn		0.008	0.012	0.027	0.026	11.000^b^	0.066
	Co		<LOD	<LOD	0.0003	0.0002	13.500^b^	0.113
	Cu		0.216	0.077	0.487	0.525	19.000^b^	0.388
	Zn		1.309	0.272	2.296	1.228	-2.366^a^	0.034*
	Se		0.007	0.004	0.036	0.034	3.000^b^	0.003*
	Mo		0.001	0.002	0.018	0.036	17.500^b^	0.272
	Sn		0.102	0.073	0.166	0.076	-1.644^a^	0.124
Bone	Ca		320,545.995	13,845.508	273,469.050	19,228.194	5.538^a^	< 0.001*
	P		110,062.456	5364.193	92,739.045	3907.156	6.768^a^	< 0.001*
	Mg		6103.294	540.307	4537.1088	343.482	6.264^a^	< 0.001*
	K		551.586	72.051	434.745	42.487	3.555^a^	0.003*
	Na		8606.449	355.414	7082.525	579.192	6.359^a^	< 0.001*
	Li		1.421	0.773	0.232	0.038	3.719^a^	0.002*
	Cr		0.131	0.234	4.148	5.472	7.000^b^	0.017*
	Mn		0.788	0.213	1.388	0.713	-2.408^a^	0.031*
	Co		0.065	0.005	0.091	0.076	22.000^b^	0.607
	Cu		0.054	0.115	0.754	0.347	< 0.001^b^	< 0.001*
	Zn		63.343	5.831	59.249	8.355	1.124^a^	0.281
	Se		0.003	< 0.001	0.006	< 0.001	−266.771^a^	< 0.001*
	Mo		0.027	0.019	0.398	0.737	< 0.001^b^	< 0.001*
	Sn		0.182	0.103	<LOD	<LOD	–	–
Body weight		kg	46.5	2.916	49.5	2.091	16.500^b^	0.224

^a^– the Student’s t-test result, ^b^– Mann-Whitney test results, M-mean, SD- standard deviation, <LOD - below the limit of detection, *statistically significant values at *p* < 0.05

### Comparison of the concentrations of the investigated minerals in tissues from wild and farmed animals

The mean concentrations of macro- and microelements in the bone marrow and bones of wild and farmed deer were compared (Table 2). The mean concentrations of K and Na in the bone marrow of the farmed animals were significantly higher than in wild deer (*p* < 0.05). The mean Zn and Se levels in the bone marrow of the farmed animals were significantly higher than in wild deer (*p* < 0.05). The mean concentrations of Ca, P, Mg, K, Na and Li in the bones of wild deer were significantly higher than in the bones of the farmed animals (*p* < 0.05). In turn, the mean concentrations of Cr, Mn, Cu, Se and Mo in the bones of the analysed animals exhibited significant differences (*p* < 0.05) and was higher in the farmed deer than in the wild animals (Table 2).

There was a significant negative correlation between the mean concentration of Ca in the bone marrow and Co and Zn content in the bones of all animals (*p* < 0.05). The mean content of K in the bone marrow was markedly correlated with that of Ca, P, Na and Li in the bones of the examined deer (*p* < 0.05). Furthermore, the mean concentration of Na in the bone marrow was significantly negatively correlated with the content of Ca, P, Mg, Na and Li and positively correlated with that of Cr, Cu, Se and Mo in the bones of the animals (*p* < 0.05). A significant negative correlation was also observed between the mean content of Se in the bone marrow and the content of Ca, P, Mg and Na in deer bones (*p* < 0.05). Significant differences were found between the mean concentration of Se in the bone marrow and those of Li, Cr, Cu, Se and Mo in the bones and between Mn content in the bone marrow and Mo level in the bones (*p* < 0.05). There was a negative correlation between Se in the bone marrow and Li in bone, and a positive correlation between Se content and those of Cr, Cu, Se and Mo as well as between Mn and Mo concentrations (*p* < 0.05) (Table 3).

**Table 3:** Comparison of the average concentration of microelements in deer bone marrow and bones

Wild and farm deer		Bone [mg/kg]												
		Ca	P	Mg	K	Na	Li	Cr	Mn	Co	Cu	Zn	Se	Mo
Bone marrow [mg/kg]	Ca	*R* = -0.289 *p* = 0.296	*R* = -0.257 *p* = 0.354	*R* = -0.304 *p* = 0.271	*R* = -0.339 *p* = 0.216	*R* = -0.221 *p* = 0.427	*R* = -0.332 *p* = 0.226	*R* = -0.136 *p* = 0.629	*R* = -0.350 *p* = 0.201	*R* = -0.539 *p* = 0.038*	*R* = 0.264 *p* = 0.341	*R* = -0.603 *p* = 0.017*	*R* = 0.257 *p* = 0.354	*R* = 0.150 *p* = 0.593
	P	*R* = -0.336 *p* = 0.221	*R* = -0.436 *P* = 0.104	*R* = -0.328 *p* = 0.232	*R* = -0.378 *p* = 0.232	*R* = -0.257 p = 0.354	*R* = -0.425 *p* = 0.114	*R* = 0.021 *p* = 0.939	*R* = -0.164 *p* = 0.558	*R* = -0.311 *p* = 0.259	*R* = 0.396 *p* = 0.143	*R* = -0.471 *p* = 0.076	*R* = 0.367 *p* = 0.177	*R* = 0.289 *p* = 0.295
	Mg	*R* = -0.296 *p* = 0.283	*R* = -0.411 *p* = 0.128	*R* = -0.253 *p* = 0.362	*R* = -0.293 *p* = 0.289	*R* = -0.214 *p* = 0.443	*R* = -0.346 *p* = 0.206	*R* = 0.050 *p* = 0.859	*R* = -0.118 *p* = 0.676	*R* = -0.164 *p* = 0.558	*R* = 0.361 *p* = 0.187	*R* = -0.275 *p* = 0.321	*R* = 0.328 *p* = 0.232	*R* = 0.250 *p* = 0.369
	K	*R* = -0.518 *p* = 0.048*	*R* = -0.589 *p* = 0.021*	*R* = -0.454 *p* = 0.089	*R* = -0.378 *p* = 0.164	*R* = -0.564 *p* = 0.028*	*R* = -0.514 *p* = 0.049*	*R* = 0.318 *p* = 0.248	*R* = -0.303 *p* = 0.271	*R* = 0.057 *p* = 0.839	*R* = 0.321 *p* = 0.243	*R* = -0.161 *p* = 0.0567	*R* = 0.250 *p* = 0.368	*R* = 0.407 *p* = 0.132
	Na	*R* = -0.721 *p* = 0.002*	*R* = -0.789 *p* < 0.001*	*R* = -0.657 *p* = 0.007*	*R* = -0.428 *p* = 0.111	*R* = -0.739 *p* = 0.001*	*R* = -0.704 *p* = 0.003*	*R* = 0.567 *p* = 0.027*	*R* = 0.457 *p* = 0.087	*R* = 0.025 *p* = 0.929	*R* = 0.596 *p* = 0.019*	*R* = -0.121 *p* = 0.666	*R* = 0.521 *p* = 0.046*	*R* = 0.614 *p* = 0.015*
	Cr	*R* = -0.254 *p* = 0.361	*R* = -0.391 *p* = 0.149	*R* = -0.127 *p* = 0.652	*R* = 0.025 *p* = 0.929	*R* = -0.268 *p* = 0.334	*r* = −0.200 *p* = 0.474	*R* = 0.302 *p* = 0.273	*R* = 0.230 *p* = 0.408	*R* = 0.455 *p* = 0.087	*R* = 0.262 *p* = 0.344	*r* = 0.278 *p* = 0.315	*R* = 0.189 *p* = 0.498	*R* = 0.314 *p* = 0.253
	Mn	*R* = -0.334 *p* = 0.223	*R* = -0.334 *p* = 0.223	*R* = -0.338 *p* = 0.217	*R* = -0.360 *p* = 0.187	*R* = -0.287 *p* = 0.299	*R* = -0.360 *p* = 0.187	*R* = 0.345 *p* = 0.207	*R* = 0.094 *p* = 0.737	*R* = 0.061 *p* = 0.826	*R* = 0.469 *p* = 0.077	*r* = −0.129 *p* = 0.644	*R* = 0.370 *p* = 0.173	*R* = 0.636 *p* = 0.010*
	Cu	*R* = -0.361 *p* = 0.187	*R* = -0.228 *p* = 0.412	*R* = -0.121 *p* = 0.666	*R* = 0.025 *p* = 0.929	*R* = -0.311 *p* = 0.259	*R* = -0.057 *p* = 0.839	*R* = 0.203 *p* = 0.466	*R* = 0.050 *p* = 0.859	*R* = 0.171 *p* = 0.541	*R* = 0.228 *p* = 0.412	*r* = 0.395 *p* = 0.144	*R* = 0.182 *p* = 0.515	*R* = 0.175 *p* = 0.532
	Zn	*R* = -0.143 *p* = 0.612	*R* = -0.289 *p* = 0.295	*R* = -0.036 *p* = 0.899	*R* = -0.146 *p* = 0.603	*R* = -0.064 *p* = 0.819	*R* = -0.157 *p* = 0.575	*R* = 0.175 *p* = 0.532	*R* = 0.003 *p* = 0.989	*R* = 0.107 *p* = 0.703	*R* = 0.300 *p* = 0.277	*r* = −0.040 *p* = 0.887	*R* = 0.250 *p* = 0.368	*R* = 0.303 *p* = 0.271
	Se	*R* = -0.746 *p* = 0.001*	*R* = -0.611 *p* = 0.015*	*R* = -0.643 *p* = 0.009*	*R* = -0.382 *p* = 0.159	*R* = -0.721 *p* = 0.002*	*R* = -0.553 *p* = 0.032*	*R* = 0.596 *p* = 0.018*	*R* = 0.303 *p* = 0.271	*R* = -0.071 *p* = 0.800	*R* = 0.667 *p* = 0.006*	*r* = 0.282 *p* = 0.307	*R* = 0.700 *p* = 0.003*	*R* = 0.596 *p* = 0.018*
	Mo	*R* = -0.404 *p* = 0.135	*R* = -0.408 *p* = 0.131	*R* = -0.312 *p* = 0.256	*R* = -0.227 *p* = 0.415	*R* = -0.403 *p* = 0.135	*R* = -0.304 *p* = 0.778	*R* = 0.063 *p* = 0.891	*R* = -0.023 *p* = 0.942	*R* = -0.059 *p* = 0.510	*R* = 0.257 *p* = 0.994	*r* = 0.451 *p* = 0.091	*R* = 0.284 *p* = 0.880	*R* = 0.170 *p* = 0.958
	Sn	*r* = −0.558 *p* = 0.031	*r* = −0.433 *p* = 0.107	*r* = −0.362 *p* = 0.184	*r* = − 0.169 *p* = 0.547	*r* = − 0.582 *p* = 0.023	*r* = − 0.300 *p* = 0.276	*r* = 0.004 *p* = 0.988	*r* = − 0.024 *p* = 0.930	*r* = − 0.124 *p* = 0.658	*r* = 0.163 *p* = 0.561	*r* = − 0.182 *p* = 0.515	*r* = 0.412 *p* = 0.127	*r* = − 0.046 *p* = 0.868

r- Pearson r correlations, R- Spearman rank-order correlations, * statistically significant values at *p* < 0.05

The concentrations of the analysed minerals were related to the body weight of the animals (supplementary data 1). The significantly higher mean content of Cr and Mn in the bone marrow of farmed deer and of Sn in the bones of wild deer was associated with a higher body weight of the animals (*p* < 0.05). In turn, the mean concentration of Se in the bone marrow of the wild animals was significantly negatively correlated with their body weight (*p* < 0.05). Moreover, the mean concentration of Mn in the bone marrow and of Mo in the bones of all animals was significantly positively correlated with their body weight (*p* < 0.05). There was no correlation between body weight and the levels of most minerals (supplementary data 1).

## Discussion

The concentration of most macro- and microelements (K, Na, Zn and Se in the bone marrow and Cr, Mn, Cu, Se and Mo in the bones) was higher in the young farmed deer, which were culled in November, when they were only half a year old. This may be associated with the richer composition of their mothers’ milk and primarily with the better nutrition status of the hinds, who were given supplements in licks. Moreover, in winter, farmed deer are generally fed concentrated fodder composed of cereal grains, which are rich in Cr, Mn, Cu, Zn, Se, and Mo [35]. As shown by Gómez et al. [5], one of the main nutritional factors influencing early growth is maternal milk production and composition; since lactation plays an important role in post-weaning growth, antler quality and the quality of other bones are influenced as well. However, the content of all macroelements, including Ca and P, which are highly important for bone formation, was higher in the bone of wild animals, although insignificantly, than in that of farmed animals, and their Ca-to-P ratio was similar in the two groups (1.5–1.6 for the bone marrow, 2.9 for the bones). The content of K and Na in the bone marrow of farmed deer was higher than in wild animals, as these elements are excreted in urine and faeces [43]. It is possible that, in contrast to farmed deer, wild animals cannot replenish losses of these minerals on an ongoing basis; hence, the lower K and Na concentrations.

Bone marrow was not previously analysed in deer, with the exception of reindeer, which are part of the traditional diet of the Sami [44]. In comparison with the results reported by Hassan et al. [44], the concentration of Cr in the bone marrow was twofold lower in the wild animals and similar in the farmed deer. The concentration of Co and Cu in the bone marrow of wild deer was at the same level as that determined in a study of reindeer, but it was substantially higher in farmed deer [44]. Cr, Co and Cu are important essential elements for humans and animals, although their excessive intake has long been associated with toxicity in mammals [35]. Cu is a component of enzymes involved in iron metabolism, and deficiency of this element causes anaemia [35, 45]. Co is a major constituent of vitamin B12 and an essential element in the synthesis of this vitamin through bacterial fermentation in ruminant animals [35, 44]. Thus, Co deficiency causes deficiency of vitamin B12 and hence macrocytic anaemia. Cr helps to maintain normal blood glucose levels [46]. The concentrations of microelements were measured in the bone marrow of red deer in this study for the first time. Cu and Co concentrations in red deer bone marrow revealed that the consumption of this tissue by humans would supply an amount of these elements below the daily safe intake established by the WHO/FAO.

In studies on wild and farmed red deer, it is important to pay attention to Se content in the bone marrow, since significant differences between its mean concentration in the bone marrow and those of Li, Cr, Cu, Se and Mo in the bones were observed. Based on livestock standards, for white-tailed deer, Se serum concentrations are considered to be deficient in the range from 0.007 to 0.060 ppm [47]. In the studied animals, Se concentration in the bone marrow was in this range. Therefore, more research should be performed on the content of this element in soil, food and deer serum, because Se deficiency not only reduces host defence mechanisms but also impairs bone metabolism, causing osteopenia and osteoarthritis [3, 48]. In New Zealand, Se deficiency in ruminants caused periodontitis, mandibular thickening and premature tooth shedding and reduced bone density [49, 50], similarly to the lesions described in huemul in Argentina [49] resulted. Besides, Se can prevent the accumulation of toxic elements [21] and influence the concentration of some microelements. Moreover, we showed that Na and Se contents correlated with each other in the two examined tissues. It is also surprising that the Na and Se content in the bone marrow correlated with the bone content of many other minerals. This suggests that the antioxidant effect of selenium could prevent osteoporosis [51] and even atherosclerosis. The results obtained by Liu et al. [52] indicated that selenite suppressed enhanced osteoblastic differentiation and vascular calcification through the inhibition of oxidative stress.

When assessing the body condition of deer, it is recommended to determine the percentage of fat in the bone marrow [53], and most studies follow this suggestion. However, the bone marrow in young animals is not only a source of fat but also a site of cell formation. The red part of the bone marrow is responsible for the health of animals. It is part of the haemic system, through which multiplied cells are distributed throughout the organism [54]. Therefore, in the future, research should be expanded to include the analysis of the amount of macro- and microelements transported through the blood. The optimal concentrations of macro- and micronutrients that should be present in healthy deer are not known. However, it seems probable that it is beneficial for their organism to have sufficient concentrations of the analysed substances to form the skeletal system and antlers, which are shed every year, and to prevent periodic osteoporosis.

The concentration of macroelements was higher in the bones of the wild animals, which may be related to a greater variety in their diet or to the instinctive search and ingestion of food with high contents of these minerals. These elements are important for the growth of antlers, which determine their later reproductive success [32, 40]. However, a greater translocation of macroelements from the bone marrow is probable in these animals, as evidenced by their lower concentration in this tissue (significantly lower only for K and Na) compared to farmed animals. The comparison of the macroelements contents in the bones revealed that the Ca, P, Mg and Na levels were lower in *Cervus elaphus* from a farm in New Zealand [55] than in farmed and wild red deer from Poland. Ca content was lower in all animals in the study conducted by Nowicka et al. [56] but similar to the levels ​​reported by Olguin et al. [40] in farmed animals. The concentration of P in the analysed deer is higher than the levels shown by Grace et al. [55], but lower than in the study conducted by Olguin et al. [40]. Mg concentration in deer analysed by Nowicka et al. [56] is similar to that of the present study and to the levels in farmed and supplemented deer reported by Olguin et al. [40]. In contrast, the content of Ca, Mg and Na in the bones of wild animals in our study is higher than the levels reported by Nowicka et al. [56] and Olguin et al. [40]. In turn, the concentration of K in the bones of wild and farmed deer from Poland is 10 times lower than in animals from New Zealand [55], but higher than in the studies conducted by Olguin et al. [40]. K is an important macroelement, as it contributes to the reduction of Ca loss via urine and to antler development by mobilising Ca from the skeleton [57]. Despite the higher concentration of K, higher concentrations of Ca were observed in the bones of wild deer as well. The same was observed in the bone marrow of farmed animals. However, a closer analysis and comparison of these tissues from wild and farmed animals showed that the higher K content in the bone marrow was accompanied by a lower Ca level in the bones of the farmed deer. This may indicate the effect of K on calcium accumulation in hard tissues.

As far as we know, the concentration of Li in deer tissues was determined in this study for the first time. The effect of lithium on small ruminants has been investigated. Some studies have shown that 41% of lithium-deficient goats die in the first year of life. In addition, skin lesions were observed in these animals [58, 59]. The deficiency of this micronutrient may have consequences in deer as well. The concentration of Li was higher in the bones of the wild animals, which may be related to the greater food variety in their diet, as trace amounts of this element are contained in tissues of the majority of higher plants. Nevertheless, its presence does not significantly affect any biological function in deer.

The concentration of Mn in the bones of farmed deer was similar to those reported by Schultz [60], Shultz et al. [61], McCullough and Ullney [62], and Demesko et al. [63], whereas a substantially lower content of this element was determined in wild deer. Manganese is very poorly absorbed in ruminants, and limited research suggests that high dietary calcium and phosphorus may reduce manganese absorption [55, 64]. Although these minerals did not interfere with Mn absorption in the farmed animals, it is possible that this occurred in the wild animals, which exhibited higher concentrations of Ca and P in their bones. Se content in the bone in both deer groups was substantially lower than that reported by Olguin et al. [40]. These differences are probably due to the absence of any supplementation in the present study or to the fact that different deer species were analysed in the two studies. Concurrently, as shown in earlier studies conducted by Tajchman et al. [22], wild animals accumulated higher amounts of arsenic, barium and lead. These observations may confirm that selenium and zinc reduce the accumulation of toxic elements [21].

As in the study conducted by Grace et al. [65], the mean mineral content in the tissues analysed in the present study declined insignificantly with the increase deer body weight. However, the content of Mn, Cu and Zn in 6-month-old deer was much lower than in older deer with higher body weight [65]. The level of Se in wild deer bone marrow decreased significantly. Se is mainly stored in muscles rather than in bones, and its deficiency leads to white-muscle disease [35]. This may be the reason why the level of Se in bone does not reflect the Se content in the diet. However, the concentrations of Cr and Mn in the bone marrow of farmed animals, that of Sn in the bones of wild deer, as well as the content of Mn in the bone marrow and that of Mo in the bones of both groups were directly proportional to the body weight of the animals. Mo is of great importance in bone remodelling, especially as it contributes to bone lengthening by affecting the epiphyseal growth plate [66]. The mean Mo concentration in the bones of the analysed animals showed significant differences and was higher in farmed deer than in wild animals. Moreover, the mean concentration of Mo in the bones of all animals was significantly positively correlated with their body weight. However, excessive dietary intake of molybdenum induces a secondary Cu deficiency. This syndrome is predominately reported in ruminants [65–67]. Post-mortem inspection carried out by a veterinarian in accordance with Regulation (We) No. 853/2004 of the European Parliament and of the Council of 29 April 2004 establishing specific hygiene rules for food of animal origin (Journal of Laws 139 of 30.4 .2004, p. 55) revealed no typical symptoms (hair depigmentation, lameness or exhaustion) of copper deficiency in the farmed and wild deer.

## Conclusions

The obtained results indicated different levels of micro- and macro-components in the body of farm and wild deer, though without clear and strong variations. Generally macro- (Ca, P, Mg, K, Na) and microelements (Li, Cr, Co, Cu, Zn, Se, Mo, Sn) accumulated in the bones of deer may derive from the bone marrow, as evidenced by their higher concentrations in hard tissues, especially in the case of macronutrients in wild deer. In comparison with the farmed deer, the bones of wild deer exhibited higher accumulation Ca, P, Mg, K, Na and Li. In turn, higher K, Na, Zn and Se levels in the bone marrow and Cr, Mn, Cu, Se and Mo in the bones were recorded in the farmed deer. There were significant differences in the mean concentration of Se in the bone marrow and of Li, Cr, Cu, Se, which may be related to the preventive action of this mineral on atherosclerosis and oxidative stress. Moreover, the concentration of Se in the bone marrow of wild deer decreased with the increase of the animals’ body weight. In turn, the concentrations of Cr and Mn in the bone marrow of the farmed deer, of Sn in the bones of the wild animals, as well as of Mn in the bone marrow and of Mo in the bones of all animals were proportional to the animals’ body weight. The higher level of macronutrients in the bones of wild deer may be related to the higher physiological importance of these minerals for life activities in the natural environment and to the limited supply of balanced food. The levels of the majority of the microelements were higher in the tissues of the farmed animals, indicating that maternal supplementation improves calves’ nutrition.

## Supplementary Information

**Additional file 1.** Table containing comparison of the concentrations of microelements in the bone marrow and bones with the body weight of the wild and farmed red deer.

## Data Availability

The datasets used and analysed during the current study available from the corresponding author on reasonable request.

## Abbreviations

Ca: Calcium
P: Phosphor
Mg: Magnesium
K: Potassium
Na: Sodium
Li: Lithium
Cr: Chromium
Mn: Manganese
Co: Cobalt
Cu: Copper
Zn: Zinc
Se: Selenium
Mo: Molybdenum
Sn: Tin
I: Iodine
LOD: The limit of detection value
