# Supplementary material for: Comparison of the accumulation of macro- and microelements in the bone marrow and bone of wild and farmed red deer (Cervus elaphus)
Source: BMC Vet Res. 2021 Oct 10;17:324. doi: 10.1186/s12917-021-03041-2 (PMC8502351; doi:10.1186/s12917-021-03041-2)
Supplement: Supplementary file 1 — Additional file 1. Table containing comparison of the concentrations of microelements in the bone marrow and bones with the body weight of the wild and farmed red deer. [file 12917_2021_3041_MOESM1_ESM.docx]

Comparison of the concentrations of microelements in the bone marrow and bones with the body weight of the wild and farmed red deer.

| **Analyzed parameters** | | **Wild red deer body weight** | | **Farm red deer body weight** | | **All** | |
| --- | --- | --- | --- | --- | --- | --- | --- |
|  |  | R^a^/r^b^ | p | R^a^/r^b^ | p | R^a^/r^b^ | p |
| Bone marrow | Ca | 0.256^a^ | 0.506 | -0.227^a^ | 0.665 | 0.007^b^ | 0.979 |
|  | P | 0.317^a^ | 0.404 | -0.094^b^ | 0.859 | 0.147^b^ | 0.600 |
|  | Mg | 0.175^b^ | 0.651 | 0.140^a^ | 0.791 | 0.131^b^ | 0.643 |
|  | K | -0.596^a^ | 0.090 | 0.467^a^ | 0.350 | 0.066^b^ | 0.815 |
|  | Na | -0.510^b^ | 0.160 | 0.486^a^ | 0.329 | 0.119^b^ | 0.694 |
|  | Li | - | - | - | - | - | - |
|  | Cr | -0.348^b^ | 0.357 | 0.828^b^ | 0.042* | 0.174^b^ | 0.533 |
|  | Mn | 0.412^b^ | 0.269 | 0.942^b^ | 0.004* | 0.628^b^ | 0.012* |
|  | Co | - | - | 0.516^b^ | 0.294 | - | - |
|  | Cu | -0.512^a^ | 0.158 | 0.600^b^ | 0.208 | 0.102^b^ | 0.717 |
|  | Zn | -0.024^a^ | 0.950 | 0.401^a^ | 0.430 | 0.216^b^ | 0.438 |
|  | Se | -0.786^b^ | 0.011* | 0.086^b^ | 0.871 | -0.084^b^ | 0.766 |
|  | Mo | -0.297^b^ | 0.436 | 0.394^b^ | 0.438 | 0.164^b^ | 0.559 |
|  | Sn | -0.611^a^ | 0.081 | 0.266^a^ | 0.610 | -0.236^a^ | 0.398 |
| Bone | Ca | 0.600^a^ | 0.088 | 0.056^a^ | 0.916 | 0.042^a^ | 0.882 |
|  | P | -0.042^a^ | 0.915 | 0.789^b^ | 0.062 | 0.017^b^ | 0.953 |
|  | Mg | -0.299^a^ | 0.433 | 0.335^a^ | 0.517 | -0.257^a^ | 0.355 |
|  | K | -0.640^a^ | 0.063 | 0.267^a^ | 0.608 | -0.0472^a^ | 0.076 |
|  | Na | 0.600^a^ | 0.088 | 0.056^a^ | 0.916 | 0.042^a^ | 0.882 |
|  | Li | -0.286^a^ | 0.456 | 0.304^a^ | 0.558 | -0.259^b^ | 0.351 |
|  | Cr | 0.125^b^ | 0.747 | 0.542^b^ | 0.265 | 0.377^b^ | 0.165 |
|  | Mn | 0.637^a^ | 0.871 | -0.015^a^ | 0.978 | 0.277^b^ | 0.317 |
|  | Co | -0.092^b^ | 0.813 | 0.371^b^ | 0.468 | 0.041^b^ | 0.884 |
|  | Cu | 0.209^b^ | 0.589 | -0.549^a^ | 0.259 | 0.347^b^ | 0.205 |
|  | Zn | -0.025^a^ | 0.950 | 0.094^a^ | 0.859 | -0.159^a^ | 0.571 |
|  | Se | -0.619^a^ | 0.075 | -0.552^a^ | 0.257 | 0.189^b^ | 0.498 |
|  | Mo | 0.518^b^ | 0.152 | 0.771^b^ | 0.072 | 0.570^b^ | 0.026* |
|  | Sn | 0.694^b^ | 0.037* | - | - | - | - |

^a^– Pearson r correlations, ^b^– Spearman rank-order correlation, * statistically significant values at *p* < 0.05
